# Rapid recovery of high content phytosterols from corn silk

**DOI:** 10.1186/s13065-017-0277-1

**Published:** 2017-10-18

**Authors:** Haiyan Zhang, Xiaowan Cao, Yong Liu, Fude Shang

**Affiliations:** 1 College of Life Science, Henan University, Dongjing Street, Jinming District, Kaifeng, Henan province 475004 People’s Republic of China; 2 College of Chemistry and Chemical Engineering, Henan University, Kaifeng, Henan 475004 China

**Keywords:** Corn silk, Phytosterols, Extraction, Purification, Crystallization

## Abstract

**Background:**

Phytosterols have important physiological and officinal function.

**Methods:**

An efficient ultrasonic assisted extraction, purification and crystallization procedure of phytosterols was established from corn silk for the first time.

**Results:**

The orthogonal test was applied to optimize the process parameters and a maximum phytosterols recovery as high as 10.5886 mg/g was achieved by ultrasonic treatment for 55 min with liquid–solid ratio of 12:1 at 35 °C, 220 w. The ultrasonic extraction temperature (T, °C) has the most significant effect on extraction yield of phytosterols. An orthogonal crystallization test was performed and the optimal conditions [crystallization temperature of 8 °C, time of 12 h and solid–liquid ratio of 1:1 (g/ml)] afforded maximum phytosterols purity of 92.76 ± 0.43%.

**Conclusions:**

An efficient extraction and crystallization procedure was established.

## Background

Steroids are one type of components in living organisms and the importance of these as a part of a healthy diet has been emphasized in the recent dietary recommendations [1]. Different functions of steroid from plants and microorganisms, such as sitosterol, stigmasterol, campesterol, ergosterol, fucosterol have been studied including inhibiting tumor growth, cellular immune stimulating, anti-inflammatory, antioxidant and anti-diabetic properties [2, 3]. The study of Andersson et al. demonstrated that sterols could be used on the prevention and treatment of the cardiovascular and cerebrovascular diseases (CCD) through higher intake of phytosterols in a natural diet to low levels of total and LDL (low density lipoprotein)-cholesterol in the serum [4]. The recommended dosage of phytosterols in sterol-enriched foods is 2 g/day, whereas the average daily intake of natural plant sterols is estimated to be 200–300 mg [5, 6]. Nowadays, while considerable progress has been made with obtaining phytosterols, the costs of it are still very high when larger amounts of pure sterols are required for structure–function studies in the nutritional, pharmaceutical and plant biology fields, thus the finding of natural materials with high content phytosterols as well as selection of a convenient isolation technology to reduce the production cost of phytosterols are very necessary.

Dried stigmata of maize (*Zea mays* L.) female flowers, commonly known as “corn silk” is distributed widely throughout the world. CS is a well-known traditional herb that has been used for treatment of varied diseases such as treating obesity [7], weight loss [8], immune enhancement [9], anti-diabetic activity [10], regulation of blood sugar [11], anti-proliferative effects on human cancer cell lines [12], improvement of gastrointestinal movement [13] and antioxidant activity [14]. Lee reported that maysin [rhamnosyl-6-C-(4-ketofucosyl)-5, 7, 3′4′-tetrahydroxyflavone], a flavone glycoside, was abundant in CS and dose-dependently reduced the PC-3 cell viability, with an 87% reduction at 200 μg/ml [15]. In spite of various pharmacological activities, corn silk is still considered as a waste during corn processing. Corn silk has been reported to contain various chemicals [14, 16, 17], however, most of the researches have been focused on the proteins, flavonoids and polysaccharides, and there is no report about the extraction of steroids from CS.

In this context, the extraction assisted with ultrasound, purification and crystallization of CS were investigated firstly to obtain an effective method for CS with higher yield. Orthogonal test method was designed to optimize the process parameters of extraction and crystallization. The aim of this study was to investigate the content of bioactive compounds-sterols in CS and establish the extraction and purification method of phytosterols in order to convert CS as waste into value-added products.

## Methods

### Materials and reagents

CS samples of two cultivars, corn silk (zhengdan 958, xianyu 335), were harvested in September 2013 in Kaifeng farmland of China, pulverized and sifted through a 80-mesh sieve to obtain the powdered samples. The CS powder was dried at 80 °C overnight and stored with dark bags in dry environment prior to the experiments.

Stigmasterol (assay purity 97%), campesterol (assay purity 98%), β-sitosterol (assay purity 97%) were all acquired from Sigma Aldrich (Sydney, Australia). All other chemicals and reagents were purchased locally and of HPLC or analytical grade. Ultrapure water was used throughout the experiments and obtained using a Millipore water purification system (Element A10).

### Preparation of the standard solution of β-sitosterol

Standard solution of β-sitosterol was prepared in anhydrous ethanol at a concentration of 100 mg/l. 2.0 ml sample after tenfold dilution was added into the tubes with 2.0 ml anhydrous ethanol and 2.0 ml ferric chlorine–strong phosphoric acid–sulfuric acid reagent, phytosterols were determined at 530 nm wavelength after 15 min reaction by sulfate–phosphate–ferric method according to Xu [18].

**Fig. 1:**
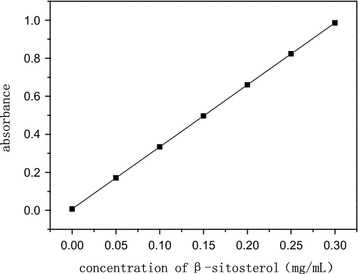
The standard curve of β-sitosterol

The regression equation is y = 3.2622x + 0.0074 (R^2^ = 0.9988), where y is the value of absorbance (mg/ml), x is the mass concentration of β-sitosterol. The good linearity could be found in the range of 0.05–0.3 mg/ml and was suitable for the determination of total phytosterols in CS (Fig. 1).

### The saponification of phytosterols from CS

5 g CS with 20 ml of a 2 M KOH in ethanol solution (95%) was weighed into the reaction triangular flask. The reaction tube was closed with parafilm and incubated at 80 °C for 2 h in water bath for the saponification. The parafilm was uncovered for the evaporation of ethanol after the saponification.

### The extraction of different solvents to phytosterols of CS

CS was extracted with petroleum ether, hexane, diethyl ether, ethyl acetate, acetone, anhydrous ethanol, butyl alcohol or trichloromethane after the saponification for 3 h [1:10 (w/v)] and centrifuged at 8000 rpm for 30 min to obtain a clear solution. The supernatant was evaporated to dryness, and reconstituted to same volume, then absorbance was measured after 15 min. Phytosterols yield (y, mg/g) is calculated using the following equation:

1 $$\text{y} = \frac{{{\text{{c}}} \times {{\text{v}}} \times {{\text{b}}}}}{\text{m}},$$

where C is the concentration of β-sitosterol calculated from the standard curve equation (mg/ml); b is the dilution factor; V is the total volume of extraction solution (ml); and *m* is the weight of raw material (g).

### Single factor and orthogonal test of ultrasonic assisted extraction on the yield of phytosterols

1 g CS in 4 ml anhydrous ethanol without the saponification was placed into the ultrasonic device. Various ultrasonic temperature from 25 to 65 °C, time from 15 to 75 min, liquid–solid ratio from 8:1 to 20:1 (ml/g), and ultrasonic power from 100 to 260 W were designed to analyze the optimal extraction of CS. Orthogonal test design with four factors and three levels (ultrasonic time, ultrasonic temperature, liquid–solid ratio, and ultrasonic power) for the phytosterols extraction from CS after the saponification was used to optimize the ultrasonic extraction parameters (Table 1).

**Table 1:** Factors and levels of orthogonal test of ultrasonic treatment

	Liquid–solid ratio (ml/g)	Time (min)	Power (W)	T (°C)
1	8:1	35	140	35
2	12:1	45	180	45
3	16:1	55	220	55

### Phytosterols purification and precipitation of CS

The extraction mixture after ultrasonic processing dried by rotary evaporation instrument was washed with hot ultrasonic water, and extracted by chloroform for three times at 55 °C. After rotary evaporation, ethyl acetate was mixed with the crude extract by slight heating, then, solution was allowed to precipitation. The solid–liquid ratio of phytosterols to ethyl acetate, crystallization temperature, and crystallization time were varied to optimize the precipitation conditions.

### Orthogonal test effect of precipitation and crystallization of phytosterols

On the data of single factor, orthogonal test method with three factors and three levels was designed to optimize the precipitation parameters (Table 2).

**Table 2:** Factors and levels of orthogonal test of precipitation

	Liquid–ratio solid (ml/g)	Time (h)	T (°C)
1	1:2	8	8
2	1:1	12	4
3	3:2	16	0

The phytosterols pellet was allowed to crystallization by methanol and then, crystal recovery was obtained by vacuum filtration using Buchner funnels and 47 mm diameter, 0.2 µm pore diameter cellulose acetate membranes supported on Whatman S42 filter paper (2.7 µm nominal pore diameter).

### Gas chromatographic analysis of phytosterols

An Shimadzu gas chromatography with flame ionisation detection (GC-FID, GC-14B) equipped with a DB-1 (30 m × 0.53 mm × 0.25 μm) capillary column was used to analyze phytosterols composition using a 1:20 split ratio injection at 280 °C with nitrogen carrier gas (purity ≥99.999%). β-sitosterol was used as an internal standard. The initial column temperature was held at 285 °C for 30 min and then increased at a rate of 10 °C/min to 300 °C and maintained for a further 5 min with a flow of 1.0 ml/min and 2 μl sample. The purity of sterols is calculated by using Eq. 2:

2 $$Y = F\frac{{{\text{A}}{_{{\text{sample}}}} \times {\text{m}}{_{{\text{internal}}}}}}{{{\text{A}}{_{{\text{internal}}}} \times {\text{m}}{_{{\text{sample}}}}}} \times 100\% ,$$

where Y is the content of sterols (%), *F* is the correction factor, A sample is the total peak area of sterols in a sample, A internal is the total peak area of internal standard, m internal is the mass of the internal standard (mg), and m sample is the mass of a sample (mg). The correction factor *F* was obtained from the standard sample (95.30% purity).

### Data analysis

All the experiments were done in triplicate, errors presented in figures are ±5% from the mean value.

## Results and discussion

Steroids have wide biological effects in living organisms, including anti-oxidative, anti-carcinogenic and anti-inflammatory activities [12, 19], their cholesterol-lowering capacities by inhibiting intestinal absorption of cholesterol have been extensively researched. Jong [20], however, the source of sterols is limited and difficult to obtain large amount of it easily. The development of new raw material with high sterols is important. CS is a waste material from corn cultivation and available in abundance throughout the world, in this paper, the extraction and purification were studied firstly and phytosterols crystal was obtained at high yield (10.5886 mg/g) and purity (92.76 ± 0.43%).

### Effect of the saponification to phytosterols of CS

**Fig. 2:**
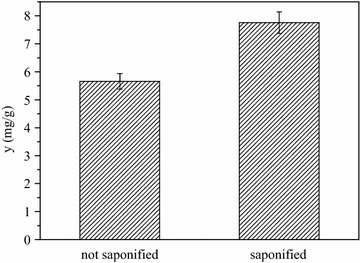
Effects of the saponification on yield of phytosterols from CS

The purpose of the saponification was to decompose the bound of sterol. The saponification of free sterol studied by Yan et al. showed that the free sterol content in the feed solution after the saponification increased from 6.64 to 9.30% as the NaOH/SODD mass ratio increased from 0.17/1 to 0.33/1 (w/w) [21]. In our study, the free phytosterols content in CS was 5.66 mg/g and increased to 7.76 mg/g in the feed solution after CS was saponified. The results indicate that the saponification can transfer the bound sterols into free sterols more efficiently, and the process is benefit to sterol recovery from CS (Fig. 2).

### Effects of extraction solvent

**Fig. 3:**
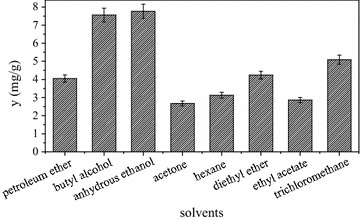
Effects of extraction solvents on yield of phytosterols from CS

As shown in Fig. 3, phytosterols extracts of CS by different organic solvents varied greatly in yield. The yield of sterol was the highest in alcohol (7.76 mg/g), then in butyl alcohol (7.56 mg/g) and lowest in acetone (2.67 mg/g), thus, alcohol was chosen as solvent to extract phytosterols from CS.

### Effects of single factor tests with ultrasonic extraction

#### Effects of ultrasonic time

**Fig. 4:**
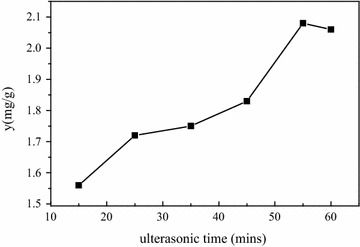
Effects of ultrasonic time on yield of phytosterols from CS

Ultrasound has great influence on the cavitation threshold, which is responsible for acoustic cavitation and also results in the formation of cavitational nucleus and been investigated to extract all kinds of materials from various plant materials [22–24]. As shown in Fig. 4, ultrasonic wave exhibited positive effect with the increase of time. The highest yield of phytosterols (2.12 mg/g) was obtained at 55 min. The time effects such as liquid circulation and turbulence produced by cavitation helped in increasing the contact surface area between the solvent and targeted compounds by permitting greater penetration of solvent into the sample matrix and thus increased the extraction efficiency [25].

#### Effects of ultrasonic temperature

**Fig. 5:**
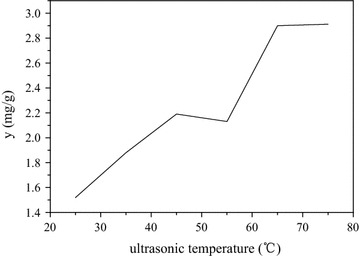
Effects of ultrasonic temperature on yield of phytosterols from CS

Due to ultrasound facilitates the disruption of CS cell wall, which enhances both the solubility and release of phytosterols to the exterior solvent, studies were conducted to evaluate the effect of ultrasound temperature over the CS [26]. From the results, it can be concluded that the content of phytosterols increased gradually along with the increase of temperature. When temperature is increased above 55 °C, density and viscosity of the extracts were decreases, which facilitate the solvent penetration deeper into sample matrix [27]. As solvent moves deeper, its area of exposure increases which ends up with higher extraction efficiency (Fig. 5). The results are in agreement with gao and Maran [24, 28], they clearly indicated that higher ultrasonic temperature enhances the extraction efficiency of phytosterols from rhizome of *Begonia grandis* Dry subsp. Sinansis (A. DC.) Irmsch. and polysaccharide of CS (Fig. 5).

#### Effects of liquid–solid ratio

**Fig. 6:**
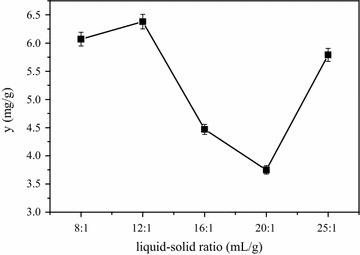
Effects of ratio of liquid–solid on yield of phytosterols from CS

It’s clear that the extraction efficiency of phytosterols increased with solid–liquid ratio ranges from 8:1 to 12:1 (ml/g) and then decreased sharply, however, it began to increase when the liquid–solid ratio increased from 16:1. Suitable high concentration of liquid–solid ratio enhances the efficiency of extraction by creating a concentration difference between the interior plant cell and the exterior solvent, which in turn augments the mass transfer rate and ends up with the increase in extraction efficiency [25]. However, a higher liquid–solid ratio may lead to some impurities in target extract which will in turn influence the purification and crystallization of phytosterols from CS and add solvent consumption. In consideration of above, the optimal extraction liquid–solid ratio of 12:1 could be selected (Fig. 6).

#### Effects of ultrasonic power

**Fig. 7:**
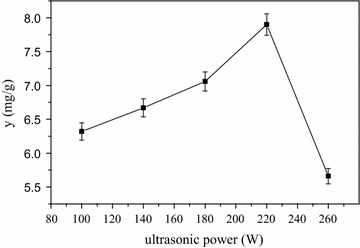
Effects of ultrasonic power on yield of phytosterols from CS

The extraction content of phytosterols increased when extraction power was increased from 100 to 220 W but decreased rapidly when the ultrasonic power was above 220 W. The results showed that 220 W power was suitable to the extraction of phytosterols from CS (Fig. 7).

### Orthogonal test effects of ultrasonic assisted extraction on the yield of phytosterols

To determine the optimal conditions and maximize the percent yield of phytosterols from CS on the data of single factor study, the effects of the four independent variables (extraction temperature of 35–65 °C, ultrasonic power of 140–220 W, time of 35–65 min and solid–liquid ratio of 1:8–1:16 g/ml) on the extraction field was investigated by employing the orthogonal test statistical experiment design with three levels. As shown in Table 3, the ultrasonic temperature had the most positive effects on the extraction yield of phytosterols, then, solid–liquid ratio had slightly significant affected, and then was extraction time, at last was ultrasonic power. By finding the optimal solution through the range analysis, the optimal extraction program was determined as follows: extraction time of 35 °C, solid–liquid ratio of 1:12, extracting time of 55 min with 220 W ultrasonic power, under these conditions, the yield of phytosterols was 10.5886 mg/g.

**Table 3:** Results of orthogonal test and range analysis

Run no.	Time (min)	Liquid–solid ratio (ml/g)	T (°C)	Power (W)	Yield (mg/g)
1	35	8:1	35	140	8.9628
2	35	12:1	45	180	8.2374
3	35	16:1	55	220	6.2627
4	45	8:1	45	220	8.0205
5	45	12:1	55	140	7.8283
6	45	16:1	35	180	7.7538
7	55	8:1	55	180	7.6910
8	55	12:1	35	220	10.5886
9	55	16:1	45	140	7.9650
K1	7.8230	8.2622	9.101	8.2520	
K2	7.8675	8.884	8.0743	7.8941	
K3	8.7475	7.2705	7.2606	8.2906	
*R*	0.9245	1.609	1.8404	0.3965	

At present, phytosterols are generally recovered from the oil deodorizer distillate (ODD) of vegetable oils [28, 29], however, the source of deodorizer distillate is limited and difficult to obtain large amount of it easily. The development of new raw material with high phytosterols is important and urgent. In prior work of Wang et al. [30], Longyan (*Dimocarpus Lour.*) seed was used as the material to extract phytosterols with ultrasound method and the field was 4. 662 mg/g. Xu et al. developed a process to recover the maximum amount of phytosterols from mulberry root bark by response surface methodology with the aid of microwave and the phytosterols yield obtained under the optimized conditions was 7.74 ± 0.12 mg/g [31]. In our context, the phytosterols yield of CS reached 10.5886 mg/g under the optimized conditions and is much greater than the field from *E. japonica* and longyan seed.

Currently, in spite of various pharmacological activities, CS is still considered as a waste from corn processing. Only in some countries, corn silk-based products such as tea, powder, are commercially available. Our study is promising for sterol recovery from CS which has high total sterol contents, furthermore, extraction of phytosterols from CS is a beneficial opportunity to convert it as waste into value-added products, especially in countries with high corn production.

### Precipitation time and temperature

The cost effective and prompt three-step purification described here afforded phytosterols with purity above 90%. The ripening temperature, and ripening time and liquid–solid ratio were varied to optimize the precipitation conditions.

A broad range of time (8, 12, 16, 20, 24 h) and temperatures (0, 4, 8, 12, 16 °C) have been tried in the literature. As shown in Table 4, the sterol yield increased when ripening time increased and tended to stable. As for temperature, optimum crystallization conditions to obtain the highest sterol yield were ripening at 8 °C. Eduardo et al. [32] suggested that brisk chilling led to low sterol yields. It is desirable to keep T ≥ −5 °C for a single-stage crystallization process. The yield of phytosterols in a 1:1 liquid–solid ratio system was much higher than that in other liquid–solid system.

**Table 4:** The influence of time and temperature and liquid–solid ratio of precipitation

Ripening time (h)	Purity (%)	Ripening temperature (°C)	Purity (%)	Liquid–solid ratio (ml/g)	Purity (%)
8	77.42	0	77.39	1:2	79.06
12	79.51	4	79.25	1:1	81.30
16	83.23	8	82.32	3:2	77.54
20	83.78	12	80.91	2:1	75.39
24	82.05	16	78.01	5:2	71.83

### Orthogonal test effects of precipitation

The application of statistical experimental design in phytosterols extraction developed closer conformance of the process output or response to target requirements and reduced process cost, development time and variability. In the present study, three factors at three levels orthogonal test were used to investigate the influence of process variables of temperature, time, and solid–liquid ratio on the precipitation of phytosterols from CS (Table 5). By analyzing these data, the optimal precipitation conditions of the extracted phytosterols lay in the following: crystallization time for 12 h, ultrasonic temperature at 8 °C, and liquid–solid ratio at 1:1.

**Table 5:** Results of orthogonal test and range analysis

Run no.	Ripening time (h)	Liquid–solid ratio (ml/g)	Ripening T (°C)	Purity (%)
1	1 (8)	1 (1:2)	1 (0)	82.9312
2	1	2 (1:1)	2 (4)	79.3256
3	1	3 (3:2)	3 (8)	85.3572
4	2 (12)	1	2	81.5346
5	2	2	3	87.6154
6	2	3	1	87.1409
7	3 (16)	1	3	83.7621
8	3	2	1	84.9156
9	3	3	2	77.5807
k	82.5380	83.1271	84.9959	
k	85.4303	83.9522	79.4803	
k	82.0861	83.3596	85.5782	
*R*	3.3442	0.8251	6.0979	

### Crystallization

According to Yan et al. [21], a variety of solvents including various alcohols, esters, ketones, alkanes, and aromatics were used in the crystallization of phytosterols from soybean ODD (oil deodorizer distillate). When ketones or alcohols were used as crystallization solvents, the yield of phytosterols decreased and no phytosterols crystals precipitated, probably due to increased solubility of sterols as the hydrocarbon chain length of alcohols and ketones increased. Methanol gave the best crystallization efficiency with high yield and high purity, so, in our study, methanol was selected to be the crystallization solvent.

Since phytosterols are insoluble in some cold solvents, they could be obtained by crystallization [33]. Rapid, cost effective and simply method of crystallization is the important strategy in phytosterols purification compared to other methods. For example, petroleum ether with water as cosolvent could generate desirable 94.7% purity during crystallization with methyl esters of soybean ODD as the extraction material. The optimal 89.7% purity of phytosterols was acquired with hexane as crystallization solvent using *E. japonica* seed as extraction material [33]. In this work, the purities of sterol samples obtained during crystallization were 92.76 ± 0.43%.

### GC analysis

**Fig. 8:**
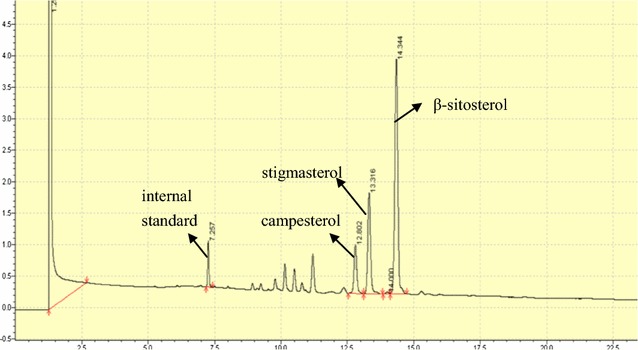
Gas chromatogram of phytosterols. All authors have the responsibility to obtain permission from the copyright holder to reproduce figures or tables that have previously been published elsewhere

**Fig. 9:**
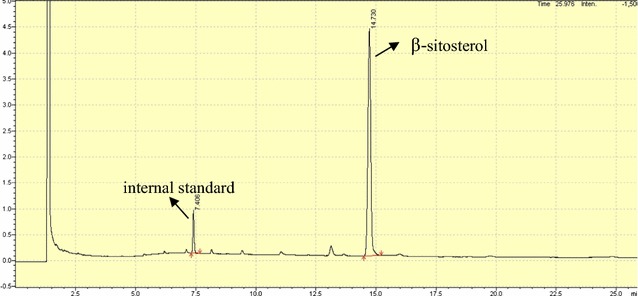
Gas chromatogram of β-sitosterol

**Fig. 10:**
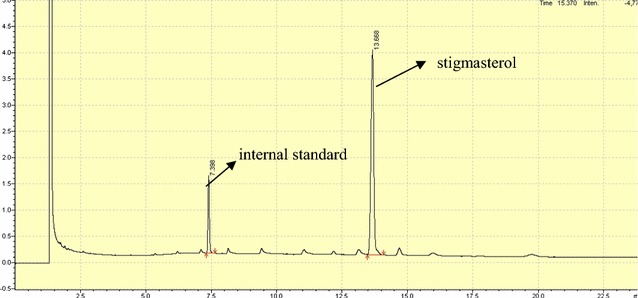
Gas chromatogram of stigmasterol

Because phytosterols are mixture, molecular weights and volatilities of various sterols are similar to each other, GC spectrometry was further utilized to determine the exact kind of sterol and content internal standard, campesterol, stigmasterol and β-sitosterol eluted with the relative retention time (RT) of 7.257, 12.802, 13.316, 14.344 min, corresponding to the relative retention time. The results showed that respectively (Figs. 8, 9, 10). According to the peak areas of sterols and Eq. 2, the purity of the recovered phytosterols sample was 92.76 ± 0.43%. In addition, GC showed that there were impurities in the sterol products; the impurities were sterol derivatives by oxidation/reduction, such as hydrogenation of sterol. The phytosterols obtained using optimal conditions contained 47.5 ± 0.24% β-sitosterol and 36.7 ± 0.13% stigosterol.

## Conclusions

In this work, the ultrasonic assisted extraction, purification and crystallization process for phytosterols recovery from CS were investigated for the first time. An efficient ultrasonic assisted extraction procedure was employed to extract phytosterols from CS for the first time. The orthogonal test was applied to optimize the process parameters of ultrasonic pretreatment (liquid–solid ratio of 12:1 for 55 min at 35 °C, 220 W) and a maximum phytosterols yield of 10.5886 mg/g could be obtained, furthermore, the conditions of purification and solvent crystallization were optimized using orthogonal test. The purity of 92.76 ± 0.43% was obtained. The process is a promising route to recover phytosterols from CS which might be a new source of natural phytosterols for health food and therapeutics with relative highly content of sterols.

## References

[1] Seal CJ (2006). Whole grains and CVD risk. Proc Nutr Soc.

[2] Jia XN, Dong W, Lu WD, Guo LZ, Wei YX (2009). In vivo immunostimulatory and tumor-inhibitory activities of polysaccharides isolated from solid-state-cultured *Trametes robiniophila* Murrill. World J Microbiol Biotechnol.

[3] Shan JJ, Zhang Y, Diao YL, Qu WS, Zhao XN (2010). Effect of an antidiabetic polysaccharide from *Inula japonica* on constipation in normal and two models of experimental constipated mice. Phytother Res.

[4] Andersson SW, Skinner J, Ellegard L, Welch AA, Bingham S, Mulligan A, Andersson H, Khaw K-T (2004). Intake of dietary plant sterols is inversely related to serum cholesterol concentration in men and women in the EPIC Norfolk population: a cross-sectional study. Eur J Clin Nutr.

[5] Normen L, Bryngelsson S, Johnsson M, Evheden P, Ellegard L, Brants H, Andersson H, Dutta P (2002). The phytosterols content of some cereal foods commonly consumed in Sweden and in the Netherlands. J Food Compos Anal.

[6] Valsta LM, Lemstro MA, Ovaskainen ML, Lampi A-M, Toivo J, Korhonen T, Piironen V (2004). Estimation of plant sterol and cholesterol intake in Finland: quality of new values and their effect on intake. Br J Nutr.

[7] Harhaji LJ, Mijatovic S, Maksimovic-Ivanic D, Stojanovic I, Momcilovic M, Maksimovic V, Tufegdzic S, Marjanovic Z, Mostarica-Stojkovic M, Vucinic Z, Stosic-Grujicic S (2008). Anti-tumor effect of *Coriolus versicolor* methanol extract against mouse B16 melanoma cells: in vitro and in vivo study. Food Chem Toxicol.

[8] Du J, Xu QT (2007). A study on mechanisms of stigma maydis polysaccharide on weight loss in experimental animals. Chin Pharmacol Bull.

[9] Kim KA, Choi SK, Choi HS (2004). Corn silk induces nitric oxide synthase in murine macrophages. Exp Mol Med.

[10] Rau O, Wurglics M, Dingermann T, Abdel-Tawab M, Schubert-Zsilavecz M (2006). Screening of herbal extracts for activation of the human peroxisome proliferator-activated receptor. Pharmazie.

[11] Zhao WZ, Yin YG, Yu ZP, Liu JB, Chen F (2012). Comparison of antidiabetic effects of polysaccharides from corn silk on normal and hyperglycemia rats. Int J Biol Macromol.

[12] Yang J, Li X, Xue Y, Wang N, Liu W (2014). Anti-hepatoma activity and mechanism of corn silk polysaccharides in H22 tumor-bearing mice. Int J Biol Macromol.

[13] Du J, Xu QT, Gao XH (2007). Effects of stigma maydis polysaccharide on gastrointestinal movement. China journal of chinese materia medica.

[14] El-Ghorab A, El-Massry KF, Shibamoto T (2007). Chemical composition of the volatile extract and antioxidant activities of the volatile and nonvolatile extracts of Egyptian corn silk (*Zea mays* L.). J Agric Food Chem.

[15] Lee J, Kim S-L, Lee S, Chung MJ, Park YI (2014). Immunostimulating activity of maysin isolated from corn silk in murine RAW 264.7 macrophages. BMB Reports.

[16] Lin M, Chu QC, Tian XH, Ye JN (2007). Determination of active ingredients in corn silk, leaf, and kernel by capillary electrophoresis with electrochemical detection. J Capillary Electrophor Microchip Technol.

[17] Velazquez DV, Xavier HS, Batista JE, Castro-Chaves C (2005). *Zea mays* L. extracts modify glomerular function and potassium urinary excretion in conscious rats. Phytomedicine.

[18] Xu XJ, Yu GZ, Chen JD (2010). Determination of total sterol in soybean sterol by sulfate–phosphate–ferric method. Chin Pharm.

[19] Zilic S, Jankovic M, Basic Z, Vancetovic J, Maksimovic V (2016). Antioxidant activity, phenolic profile, chlorophyll and mineral matter content of corn silk (*Zea mays* L): comparison with medicinal herbs. J Cereal Sci.

[20] Jong N, Plat J, Mensink RP (2003). Metabolic effects of plant sterols and stanols. J Nutr Biochem.

[21] Yan F, Yang HJ, Li JX, Wang HL (2012). Optimization of phytosterols recovery from soybean oil deodorizer distillate. J Am Oil Chem Soc.

[22] Yang CX, He N, Ling XP, Ye ML, Zhang CX, Shao WY (2008). The isolation and characterization of polysaccharides from longan pulp. Sep Purif Technol.

[23] Sun YD, Liu D, Chen J, Ye X, Yu D (2011). Effects of different factors of ultrasound treatment on the extraction yield of the all-trans-carotene from citrus peels. Ultrason Sonochem.

[24] Prakash Maran J, Manikandan S, Thirugnanasambandham K, Vigna Nivetha C, Dinesh R (2013). Box-Behnken design based statistical modeling for ultrasound-assisted extraction of corn silk polysaccharide. Carbohyd Polym.

[25] Romdhane M, Gourdon C (2002). Investigation in solid–liquid extraction: influence of ultrasound. Chem Eng J.

[26] Toma M, Vinatoru M, Paniwnyk L, Mason TJ (2001). Investigation of the effects of ultrasound on vegetal tissues during solvent extraction. Ultrason Sonochem.

[27] Chen W, Huang Y, Qi J, Tang M, Zheng Y, Zhao S (2012). Optimization of ultrasound-assisted extraction of phenolic compounds from areca husk. J Food Process Preserv.

[28] Gao L, huang XJ, Ling JY (2012). Study on ultrasonic extraction technology of sterol from the rhizome of *Begonia grandis* Dry subsp. Sinansis (A. DC.) Irmsch. Med plant.

[29] Lin KM, Koseoglu SS (2003). Separation of sterols from deodorizer distillate by crystallization. J Food Lipids.

[30] Wang F, Wang J, Fu XJ (2011). Study on extraction methods of total sterol from longan seeds. J Anhui Agric Sci.

[31] Wang LJ (2015). Optimization of extraction and purification of phytosterols from eriobotrya japonica seed by response surface methodology. Food Ind.

[32] Moreira Eduardo A, Miguel A (2004). Baltanás recovery of phytosterols from sunflower oil deodorizer distillates. JAOCS.

[33] Shimada Y, Nakai S, Suenaga M, Sugihara A, Kitano M, Tominaga Y (2000). Facile purification of tocopherols from soybean oil deodorizer distillate in high yield using lipase. J Am Oil Chem Soc.

